# Effects of berberine on acquisition and reinstatement of morphine-induced conditioned place preference in mice

**Published:** 2016

**Authors:** Faezeh Vahdati Hassani, Mahmoud Hashemzaei, Edris Akbari, Mohsen Imenshahidi, Hossein Hosseinzadeh

**Affiliations:** 1 *School of Pharmacy, Mashhad University of Medical Sciences, Mashhad, Iran*; 2 *Department of Pharmacology and Toxicology, School of Pharmacy, Zabol University of Medical Sciences, Zabol, Iran*; 3 *Pharmaceutical Research Center, Department of Pharmacodynamics and Toxicology, School of Pharmacy, Mashhad University of Medical Sciences, Mashhad, Iran*

**Keywords:** *Berberis vulgaris*, *Berberine*, *Conditioned Place Preference*, *Morphine*, *Mouse*

## Abstract

**Objective:**

It has been shown that berberine, a major component of *Berberis vulgaris* extract, modulates the activity of several neurotransmitter systems including dopamine (Da) and N-methyl-D-aspartate (NMDA) contributing to rewarding and reinforcing effects of morphine. Drug craving and relapsing even after a long time of abstinence therapy are the most important problems of addiction. In the present study, we investigated the alleviating effects of berberine on the acquisition and reinstatement of morphine-induced conditioned place preference (CPP) in mice.

**Materials and Methods:**

In male NMRI mice, the acquisition of CPP was established by 40 mg/kg of morphine sulphate injection and extinguished after the extinction training and reinstated by a 10 mg/kg injection of morphine. The effects of different doses of berberine (5, 10, and 20 mg/kg) on the acquisition and reinstatement induced by morphine were evaluated in a conditioned place preference test.

**Results:**

The results showed that intraperitoneal administration of berberine (5, 10, and 20 mg/kg) did not induce conditioned appetitive or aversive effects. Injection of berberine (10 and 20 mg/kg) 2 h before the morphine administration reduced acquisition of morphine-induced CPP. In addition, same doses of berberine significantly prevented the reinstatement of morphine-induced CPP.

**Conclusion:**

These results suggest that berberine can reduce the acquisition and reinstatement of morphine-induced conditioned place preference and may be useful in treatment of morphine addiction.

## Introduction

Drug craving is a major clinical problem in drug addicts which motivates them to relapse to drug seeking behavior even long after detoxification (Ribeiro Do Couto et al., 2005 ▶). Unfortunately, there is no effective treatment available for drug addiction. It is known that DA system is implicated in mediating psychological dependency and desiring effects of morphine (Wise, 2002 ▶). Continuing the drug abuse is due to hyperactivation of mesolimbic DA system which plays a critical role in mediating the positive reinforcing effects of opiates (Lee al., 2011 ▶; Popik and Kolasiewicz, 1999 ▶). It has appeared that dopamine antagonists such as ‎haloperidol, clozapine, risperidone, and SCH 23390 show inhibitory effects on morphine-induced CPP in mice (Manzanedo et al., 2001 ▶). Moreover, GABA (gamma-aminobutyric acid) ergic inhibitory effects were inhibited following the activation of DA neurons in the ventral tegmental area (VTA) by morphine, as a result of DA transmission increase in the nucleus accumbens (NAcc) (Ribeiro Do Couto et al., 2005 ▶). Glutamatergic projections from the prefrontal cortex and amygdale onto the mesolimbic reward pathway (NAcc and VTA) have a role in modulation of dopamine neurons which originate from VTA and project to NAcc and other forebrain regions. These interactions between DA and glutamate systems are critical in development of the rewarding effects of opioids (Aguilar et al., 2009 ▶; Zhu et al., 2006 ▶). The acquisition of morphine induced CPP and sensitization of the rewarding effects of morphine are blocked by NMDA antagonists such as AP-5, MK-801, and memantine, suggesting a possible role in the treatment of drug addiction (Aguilar et al., 2009 ▶; Dallimore et al., 2006 ▶; Harris et al., 2004 ▶; Ribeiro Do Couto et al., 2005 ▶). 

*Berberis vulgaris* L. (Barberry) belongs to Berberidaceae family. The various parts of this plant including the root, bark, leaf, and fruit have been used in folk medicine. Studies suggest pharmacological effects for *B. vulgaris* or its active constituents specially berberine, an isoquinoline alkaloid, that belongs to the structural class of protoberberines (Imanshahidi and Hosseinzadeh, 2008 ▶). Berberine is reported to have therapeutic potential in a variety of central nervous system (CNS) disorders. It possesses anxiolytic, antidepressant, antipsychotic, anticonvulsant (Bhutada et al., 2010 ▶), and anti-amnesic (peng et al., 1997) activities as well as inhibitory effects on morphine-induced sensitization through reduction of DA and NMDA receptor bindings in the cortex (Chu et al., 2008 ▶; Yoo et al., 2006 ▶). So, the preventive effects of berberine on morphine-induced conditioned place preference (CPP) and locomotor hyperactivity might be due to the inhibitory influence on DA and NMDA receptors. The present work has evaluated the effects of berberine on acquisition, extinction, and reinstatement of morphine-induced CPP.

## Materials and Methods

**Animals**

Male NMRI mice, weighing 25-30 g, were obtained from the animal house, School of Pharmacy, Mashhad University of Medical Sciences, Iran. The animals were maintained in colony room under 12 h: 12 h light-dark cycle, constant temperature of 22±2 °C and 40-50% humidity conditions (4 mice per cage) and had free access to water and food. All of the treatments were carried out according to Mashhad University of Medical Sciences, Ethical Committee Acts (88336).

**Chemicals**

Morphine sulphate obtained from Darou Pakhsh, Iran, and Berberine chlorides from Sigma-Aldrich, Germany, were used in the present study. Chemicals dissolved in 0.9% normal saline were administered intraperitoneally (i.p.) in a volume of 10 ml/kg. All solutions were prepared freshly.

**Conditioned place preference (CPP) procedure**

**Apparatus**

The CPP experiment was performed in a special apparatus composed of three chambers made of plexiglas with different visual, olfactory, and tactile cues. Two chambers were of the equal size (30 cm × 30 cm × 35 cm). One had white walls with a rough floor (wide grid) and the other had black walls with a smooth floor (fine grid). Both chambers were connected by a smaller (15 cm × 30 cm × 35 cm) central, grey chamber. All chambers were separated by guillotine doors. The olfactory cues were banana essence and acetic acid from which a drop respectively was placed at the corner of the white and black chamber floors. In order to preclude any interference from the smell of feces and urine, the compartments were thoroughly cleaned following each test. 

**Experimental procedure**

The treatment schedule (Imenshahidi et al., 2014) for acquisition and extinction of morphine-induced CPP is summarized in table 1.

**Table 1:** Treatment schedule for conditioned place preference experiment

**Acquisition of place preference**			**Ext phase**	**Rein phase**
Pre (3 days)	Con (4 days)	Post (1 day)	7 days	1 day
	S+ S, S +M 40			S + M 10

S: normal saline 10 ml/kg, M: morphine, 10: 10 mg/kg, 40: 40 mg/kg,

Pre: preconditioning, Con: conditioning, Post: post-conditioning, Rein: reinstatement, Ext: extinction

**Acquisition of place preference**

**Pre-conditioning phase**

In the first phase, the mice were placed individually in the central chamber, with the doors kept open and allowed to access both chambers of the apparatus for 15 min (900 s) for 2 consecutive days. On day 3, the time spent in each chamber was recorded. The animals which spent more than 500 sec in the white or more than 400 sec in the central grey chamber were chosen for the rest of the study.

**Conditioning phase**

In the second phase which lasted 4 days, mice (n =7) were injected i.p. with normal saline and placed for an hour in the black chamber. After an interval of 4 h, the mice received morphine/berberine and were placed for one hour inside the white chamber. In the conditioning phase, the mice were divided into 8 groups as following: normal saline (S) + S, S + morphine (M) 40 mg/kg, S+ berberine (B) 5, 10 and 20 mg/kg, M 40 mg/kg + B 5, 10 and 20 mg/kg. All drugs were administrated immediately before confinement in the white compartment.

**Post-conditioning phase**

During the third phase on day 8, the mice were placed in the central chamber. The guillotine doors separating the two chambers were removed, so the mice could move freely between the chambers. The total time that each mouse spent in each chamber was recorded during a 900 s period of observation. During this phase animals did not receive any treatments. The time spent by each mouse ‎in the central chamber was divided equally between black and white chambers. The degree of drug induced conditioning place preference is calculated as the time spent during the preconditioning phase – the time spent during the post-conditioning phase in the white compartment (drug-paired compartment). If the difference was positive, the drug has induced a preference while the opposite indicates the aversion.

**Extinction of place preference**

After the post-conditioning phase, in some groups, the mice were placed in the CPP apparatus for a period of 900 s daily with the guillotine doors open. This schedule was repeated for 7 days to eliminate morphine dependency. In other words, extinction of place preference is acquired when the difference between the time spent in the white compartment in the extinction phase and pre-conditioning phase is not significant.

**Reinstatement of place preference**

After the extinction phase, on the day 16, mice were placed inside the CPP apparatus for 900 s in order to induce morphine reinstatement. The time spent in the black and white chambers was recorded. The animals in each group were injected with the respective drug or normal saline (S + M 10 mg/kg and M 10 mg/kg + B 5, 10, and 20mg/kg and tested 30 min after drug administration. The time spent in each chamber was recorded during 900s. 

**Statistical analysis**

Data were analyzed using one-way analysis of variance (ANOVA) followed by Tukey's‎ post-hoc test by ‎ GraphPad InStat version 3.00 (GraphPad Software, San Diego, California, USA). All data were presented as mean ± S.E.M. P values less than 0.05 were considered to be statistically significant.

## Results

**Effect of berberine on place preference**

**Figure 1:**
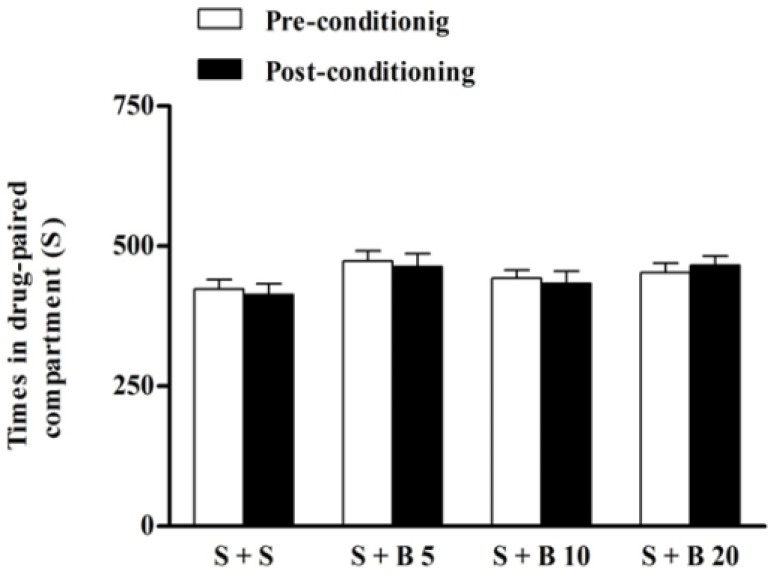
Effects of berberine alone on place preference. During the phase of conditioning, mice were treated with the following treatments in the drug-paired compartment: normal saline (S) (vehicle) + S, S+ berberine (B) 5, 10 and 20 mg/kg. Each value was expressed as mean±S.E.M (n = 7

As shown in figure 1, berberine (5, 10, and 20 mg/kg) alone compared to control (normal saline) group did not have any effects on the time spent in drug paired compartment. Accordingly, berberine did not produce conditioned place preference or conditioned place aversion (p> 0.05).

**Effect of berberine on the acquisition of morphine-induced CPP**

**Figure 2:**
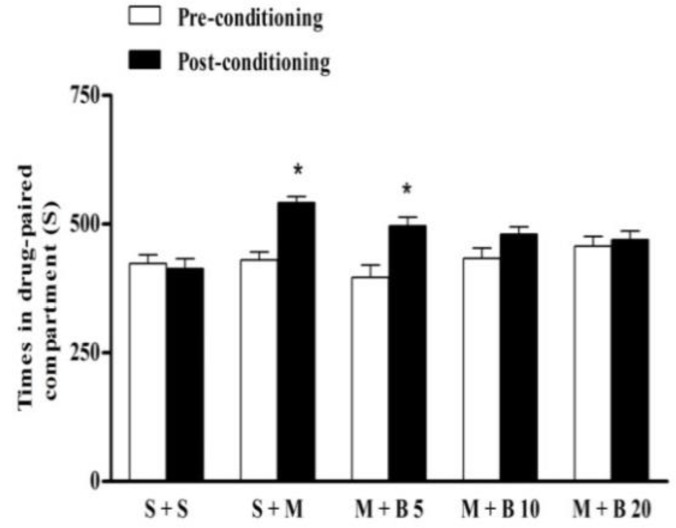
Effects of berberine on morphine-induced conditioned place preference. During the phase of conditioning, mice were treated with the following treatments in the drug-paired compartment: normal saline (S) (vehicle) + S, S+ morphine (M) 40 mg/kg, M 40 mg/kg + berberine (B) 5, 10 and 20 mg/kg. Each value was expressed as mean±S.E.M (n = 7), *p<0. 05 significant differences in the time spent in the drug-paired compartment in pre-conditioning vs. post-conditioning session tests

As shown in figure 2, the time spent in the white compartment in post-conditioning (day 8) was higher in the mice which received morphine (40 mg/kg) compared to the normal saline control group indicating morphine established CPP (P<0.05). Berberine at dose of 5 mg/kg did not affect the acquisition of morphine-induced CPP. The time spent in the white compartment (drug-paired compartment) in the post-conditioning phase (day 8) was significantly higher than the time spent in the white compartment in the pre- conditioning phase (day 3) (p<0.05). However, at dose of 10 and 20 mg/kg, there is no significant difference between the time spent in pre- and post-conditioning days (p>0.05). It showed that at higher doses berberine could prevent the acquisition of morphine-induced CPP.

**Effect of berberine on the extinction and reinstatement of morphine-induced CPP**

**Figure 3:**
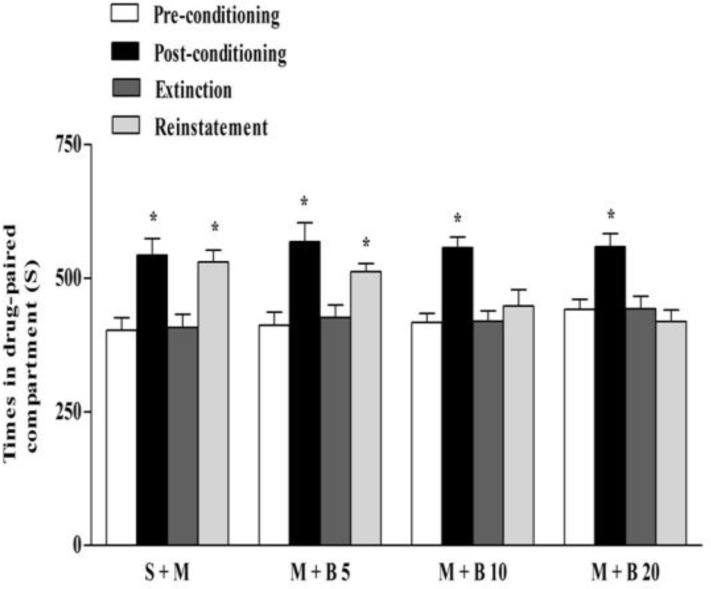
Effects of berberine on extinction and reinstatement of morphine-induced CPP. After acquisition and extinction of morphine-induced CPP, during reinstatement phase, a morphine reminding dose injected to animals in the following groups : normal saline (S) (vehicle) + morphine (M) 10 mg/kg; M 10 mg/kg + berberine (B) 5, 10 and 20 mg/kg. Each value was expressed as mean ± S.E.M (n = 7), *p<0.05 significant differences in the time spent in the drug-paired compartment in pre-conditioning vs. post-conditioning session tests or vs. reinstatement tests

In morphine receiving group (40 mg/kg) after 7 consecutive extinction days, the conditioning was lost and place preference was recovered after the administration of morphine in the reinstatement session. Injection of berberine at doses of 10 and 20 mg/kg could block the reinstatement of place preference as the difference between the time spent in the drug-paired compartment on day 16 (reinstatement) and day 3 (preconditioning) was not significant (p>0.05) (figure 3).

## Discussion

The present study evaluated the effect of berberine as a DA and NMDA inhibitor on the extinction and reinstatement of morphine-induced CPP. The results indicated that berberine induced neither conditioned place preference nor conditioned place aversion. Administration of berberine at doses of 10 and 20 mg/kg before exposure to a morphine paired compartment significantly decreased morphine-induced conditional preference and prevented morphine CPP reinstatement induced by single injection of reminding morphine on day 16. 

The previous studies reported that morphine at dose of 40 mg/kg was a powerful agent to induce CPP. This condition lasts approximately 1 week when the animals are exposed to the CPP apparatus daily for an extinction phase. Then, if the animal is exposed to an injection of 5-40 mg/kg of morphine, the CPP will reinstate (Ribeiro Do Couto et al., 2003 ▶). In the present study, administration of 10 mg/kg of morphine reinstated the morphine-induced CPP like the previous studies which used CPP model to assess the relapsing to drug-induced addiction after an extinction period (Imenshahidi et al.; 2011 ▶, Imenshahidi et al. 2014 ▶; Mueller et al., 2002 ▶). It was reported that berberine reduced D1 and NMDA receptor bindings in the cortex (Yoo et al., 2006 ▶) and inhibited catecholamine biosynthesis, e.g. DA synthesis in in vitro neuronal cells (Shin et al., 2000 ▶). A large body of evidence indicated that dopaminergic, GABAergic, and glutamatergic neuronal pathways and many brain regions such as VTA, NAcc, amygdala, and hippocampus are involved in the rewarding effects of opiates (Cami and Farre, 2003 ▶). The mesolimbic DA system is a major neurotransmitter involved in the rewarding effects produced by morphine. Administration of morphine increases the extracellular DA in the NAcc, so it is hypothesized that this stimulation of dopaminergic system plays a crucial role in the liability of abuse of drugs. It has been shown that different dopamine antagonists with different receptor blockade profiles like haloperidol and clozapine produce conditioned place aversion (Manzanedo et al., 2001 ▶). Glutamatergic neurotransmission has also a role in the acute opioid rewarding effects, development and expression of behavioral and neurochemical sensitisation to opioids (Pierce and Kalivas, 1997 ▶; Ribeiro Do Couto et al., 2004 ▶). Different biochemical studies have indicated that the DA release is regulated by glutamate and NMDA receptors (Kalivas et al., 1989 ▶, Kretschmer, 1999 ▶). Do Couto et al, 2005, demonstrated that drug-induced reinstatement of place preference may be largely independent of dopamine and more closely related to glutamatergic neurotransmission (Ribeiro Do Couto et al., 2005 ▶). Inhibition of the GABAergic inhibitory interneurons which subsequently increases the DA transmission to the NAcc by opiates leads to the activation of DA neurons in VTA (Leone et al., 1991 ▶; Johnson and North, 1992 ▶). GABA is an inhibitory neurotransmitter in the CNS and modulation of GABAergic transmission was effective against drug seeking and relapses. Baclofen, a GABAB receptor agonist, could block the expression of morphine induced CPP by promoting the extinction and preventing stress-induced reinstatement of morphine preference (Meng et al., 2014 ▶). Although studies showed that *B. vulgaris* extract may be useful in convulsion and epilepsy (Fatehi et al., 2005 ▶), berberine is not effective against pentylenetetrazole (a GABA inhibitor) -induced convulsions in mice (Bhutada et al., 2010 ▶).
